# Resting HRV predicts cardiac vagal control during stress, not psychological distress

**DOI:** 10.1038/s41598-026-52956-z

**Published:** 2026-05-23

**Authors:** Pierre Csigai, Laurent Sparrow, Léa Maquet, Aurélie Pique, Paul Kozieja, Lucas De Zorzi, Henrique Sequeira

**Affiliations:** 1 Univ. Lille, CNRS, UMR 9193 - SCALab - Sciences Cognitives et Sciences Affectives, Lille, F-59000 France; 2 Univ. Lille, CNRS, FR 2052 - SCV - Sciences et Cultures du Visuel, Tourcoing F-59200, France; 3 Univ. Lille, Department of Biology, Faculty of Sciences and Technology, Lille, F-59650, France

**Keywords:** Virtual reality, Trier social stress test, Heart rate variability, Autonomic space model, Stress reactivity, Emotional regulation, Health care, Neuroscience, Physiology, Psychology, Psychology

## Abstract

Resting heart rate variability (HRV) is considered a marker of individuals’ capacity to adapt to environmental demands, although direct empirical evidence remains limited. This study examined whether resting HRV predicts subjective and cardiac reactivity to stress, psychological distress, and emotional regulation (ER) strategies. Eighty-five healthy young adults completed distress (DASS) and cognitive ER (CERQ) scales before undergoing a virtual reality–adapted Trier Social Stress Test. Heart rate (HR), HRV indexed by RMSSD, skin conductance, and subjective stress were recorded across baseline, stress, and recovery. As expected, tasks elicited subjective and autonomic stress responses, and higher resting RMSSD predicted greater RMSSD reactivity and recovery. Contrary to our hypothesis, Bayesian analyses provided evidence for the absence of association between resting RMSSD and HR reactivity and recovery, subjective stress reactivity, psychological distress, and ER strategies. Overall, our findings challenge the view of resting HRV as a reliable index of adaptive capacity to acute stress or of vulnerability to psychological distress. Instead, resting HRV interpretation should be restricted to a predictor of the degree to which the vagal system can modulate cardiac activity. Furthermore, resting HRV’s health relevance is likely more tied to long-term cardiovascular vulnerability in the context of repeated or chronic stress.

**Supplementary Information:**

The online version contains supplementary material available at 10.1038/s41598-026-52956-z.

## Introduction

Stress involves a coordinated set of physiological responses to environmental challenges that disrupt or threaten homeostatic equilibrium^1^. Although these responses play a pivotal adaptive role when their timing and intensity are appropriately regulated, adverse consequences may arise when stress becomes excessive or chronic^2^. In such cases, the cumulative strain on the body, known as “allostatic load”, reflects the physiological “wear and tear” caused by repeated or inappropriate stress^3^. Understanding and targeting the mechanisms underlying both stress regulation and dysregulation may thus provide valuable avenues for improving mental and physical health.

A key feature of stress regulation is the capacity to dynamically adjust cardiac activity, particularly heart rate (HR), in response to changing metabolic demands. This adaptive process is primarily mediated by the autonomic nervous system (ANS), which modulates this activity through its sympathetic and parasympathetic branches. Traditionally, these branches were considered to operate in a reciprocal manner, one withdrawing as the other became active^4^. However, subsequent findings challenged this view by revealing substantial interindividual variability in autonomic response modes. Beyond reciprocal patterns, coactive patterns can occur when both branches activate or inhibit simultaneously, and independent patterns emerge when only one branch changes while the other remains stable^5^. This has prompted efforts to assess the specific contribution of each ANS branch, to determine whether particular autonomic response patterns better support cardiac adjustment to changing internal and external demands.

Among cardiac measures, heart rate variability (HRV) is particularly suited to address this question. HRV refers to the variation in time intervals between successive heartbeats and can be quantified using both time-domain and frequency-domain methods. Specifically, high-frequency components of HRV, namely the root mean square of successive differences (RMSSD) and high-frequency HRV (HF-HRV), can be considered specific indices of parasympathetic (vagal) modulation of cardiac activity^6^. When interpreted in conjunction with heart rate (HR), which reflects the integrated influence of sympathetic and parasympathetic activity, these indices provide an indirect estimate of cardiac autonomic regulation. Generally, acute stress is associated with sympathetic activation and parasympathetic withdrawal, reflected in a transient increase in HR and reductions in high-frequency HRV components. Furthermore, higher resting HRV has been associated with increased vagally mediated cardiac modulation, which has been interpreted as reflecting a greater capacity to dynamically adjust cardiac activity in response to changing environmental demands^7–9^, and lower vulnerability to chronic stress^10^.

Beyond its role as an index of autonomic cardiac regulation, HRV has increasingly been interpreted as a broader marker of individual differences in the capacity to adapt to environmental demands^7,11,12^. This conceptual extension is supported by evidence linking resting HRV not only to physiological outcomes but also to psychological functioning: lower resting HRV and greater reductions in HRV during stress have been associated with internalizing and externalizing symptoms^13^, while higher resting HRV has been linked to the use of more adaptive coping strategies^14^, including adaptive emotional regulation (ER) strategies^15^. ER can be broadly defined as the automatic and deliberate processes through which individuals influence their emotional experience and expression^16^. Among these processes, ER strategies are particularly relevant in the context of stress, which is often accompanied by, or even generated by, negative subjective experiences shaped by voluntary or intrusive thoughts. However, as these strategies vary in their short-term effectiveness and long-term consequences, rigid or repeated reliance on maladaptive forms of regulation may sustain stress, promote psychological distress, and contribute to psychopathology^17,18^.

In summary, HRV is now commonly presented as a marker of an individual’s capacity to respond adaptively to stressful situations, both physiologically and psychologically. However, direct empirical evidence examining how resting HRV relates to actual stress responses, physiological and psychological, remains limited. In this context, the aim of this study was twofold. *First*, to examine the association between HRV and cardiac stress response. *Second*, to explore the associations between resting HRV, subjective reactivity to stress, ER strategies, and psychological distress.

To this end, healthy participants were exposed to a virtual reality (VR) stress protocol adapted from the Trier Social Stress Test^17^(TSST) during which both autonomic activity and subjective stress were collected. In addition to cardiac measures, electrodermal activity was recorded as a complementary index of sympathetic activation and physiological arousal, providing a more comprehensive assessment of stress responses^19^. Furthermore, given that HRV, particularly its high-frequency components, can be influenced by respiration, respiratory activity was also monitored and accounted for in the analyses^6^. VR was chosen for its ability to reduce administration costs while improving procedural standardization, without compromising the task’s effectiveness^20^. In line with the TSST-VR, the protocol consisted of two tasks, a speech and an arithmetic task, designed to elicit psychosocial stress through performance in front of an unresponsive virtual audience. We also introduce a recovery period between tasks, making it particularly suited to examining variations in autonomic and subjective responses.

We hypothesized that (1) the tasks would elicit autonomic and subjective stress responses across participants; (2) Higher resting HRV would be associated with greater HRV and HR reactivity and recovery; (3) Higher resting HRV would be associated with lower subjective stress reactivity, greater use of adaptive ER strategies and lower psychological distress.

## Materials and methods

### Participants

Ninety-two healthy volunteers (46 women, 46 men) were recruited through social network announcements. Inclusion criteria were being between 18 and 35 years old, being a French speaker, and having normal or corrected-to-normal vision (Snellen, 1862). Exclusion criteria included any self-reported history of neurological, psychiatric, or cardiovascular disorders, as well as the use of psychoactive substances. Participants were instructed to refrain from smoking and caffeine consumption on the morning of the experiment, and compliance was verified via self-report before the session.

The required sample size was determined a priori using G*Power^21^ for the expected associations between resting RMSSD and psychometric variables (subjective stress reactivity, psychological distress, and emotion regulation). A two-tailed Pearson correlation was specified (ρ = 0.30, α = 0.05, (1-β) = 0.80), yielding a minimum of 84 participants. To account for potential data loss, particularly due to movement artifacts during speech, we increased the target sample by 10%, resulting in 92 participants. In addition, to ensure adequate statistical power for the hierarchical multiple regression analyses, we conducted a *post hoc* sensitivity analysis (three-step; f² = 0.24, α = 0.05, *N* = 84, one tested predictor among seven total predictors), which indicated high statistical power (1–β = 0.99).

Seven individuals were subsequently excluded from analyses: three (including one woman) due to ineligibility identified during anamnesis, and four (including two women) due to technical recording failures, leaving a final sample of 85 participants (43 women; mean age = 21.93 ± 3.03 years).

All participants provided written informed consent after receiving a detailed description of the study’s objectives and procedures. The study was not preregistered and was approved by the Université de Lille Ethics Committee [Ref: 2023-727-S120] and conducted in accordance with the Declaration of Helsinki at the Faculté des Sciences et Technologies, Université de Lille, France.

### Apparatus and virtual environment

Testing took place in a quiet room with moderate light. The stress protocol, developed and run on Unity (v2023.2.0), was delivered via an HTC Vive Pro Eye headset (1440 × 1600 pixels per eye, 90 Hz refresh rate, 110° field of view). The headset was connected to a DELL Precision 7560 workstation (Windows 11 Pro).

**Fig. 1:**
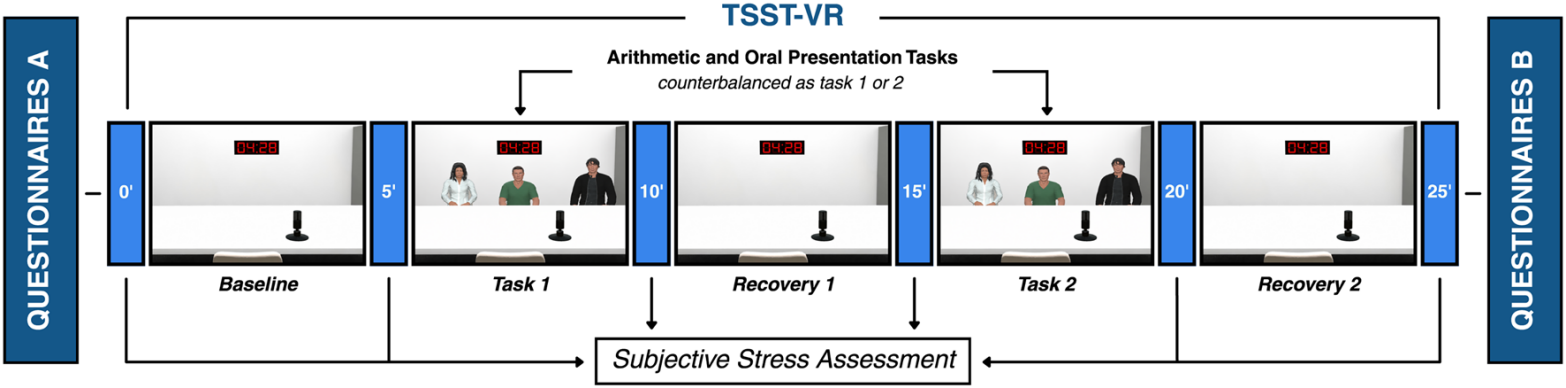
Illustration of experimental design. The procedure consisted of three stages: an initial psychometric assessment with questionnaires, the TSST-VR session structured into five 5-minute periods (baseline, task 1, recovery 1, task 2, recovery 2), and a final psychometric evaluation. Instructions and stress assessment take place at the beginning and end of baseline and recovery periods.

The virtual environment was largely inspired by previous TSST-VR implementations and recreated a standard job interview setting (cf. Figure 1). Ambient sounds (e.g., ventilation noise, ticking clock) were included to enhance immersion^22^. Participants were seated at a table equipped with a microphone and a virtual tablet used to display task instructions and collect subjective stress ratings. A digital clock positioned in front of them remained inactive during baseline and recovery periods but displayed countdowns or task-related digits during active periods. During these periods, corresponding to tasks, three unresponsive avatars, one woman and two men, dressed in everyday clothing, were seated in front of the participants.

### Measures

#### Psychometric scales

Psychological distress was assessed using a French version of the *Depression*,* Anxiety*,* and Stress Scale* (DASS-42)^23,24^ allowing the assessment of depression, anxiety, and stress symptoms over the past week. A Likert scales range from 0 (*Did not apply to me at all*) to 3 (*Applied to me very much, or most of the time*), enabling the derivation of three sub-scores associated with separate subscales (depression, anxiety, stress), each comprising fourteen items, i.e., forty-two items in total.

A French version of the *Cognitive Emotional Regulation Questionnaire* (CERQ)^25,26^ was used to assess participants’ habitual use of ER strategies in response to adverse life events. Responses were rated on a 5-point Likert scale ranging from 1 (*Almost never*) to 5 (*Almost always*). The questionnaire includes nine strategies, five considered adaptive (acceptance, positive refocusing, refocus on planning, putting into perspective, and positive reappraisal), and four considered maladaptive (self-blame, rumination, catastrophizing, and other-blame). Each strategy was assessed using four items, for a total of 36 items.

A French version of the *Liebowitz Social Anxiety Scale* (LSAS)^27,28^ was used to assess social anxiety. Through this scale, the participant rated his level of anxiety from 0 (*none*) to 3 (*severe*) and avoidance tendencies from 0 (*never*) to 3 (*usually*) when faced with twenty-four distinct social situations.

Given its impact on HRV^29^, weekly physical activity levels were assessed using the Ricci & Gagnon questionnaire^30^. The scale is structured into three sections, allowing for the measurement of sedentary behaviors, leisure-based physical activities, and daily physical activity levels.

A French version of the *Presence Questionnaire* (PQ)^31,32^ was used to assess the sense of presence within VR. This scale consists of nineteen items designed to gauge four factors contributing to the sense of presence as outlined by Witmer & Singer^32^: level of control, sensory aspects, elements of distraction, and realism.

Internal consistency for all scales was assessed in the present sample (*N* = 85) using Cronbach’s alpha and McDonald’s omega (see Results section).

#### Behavioral responses

Subjective stress was assessed before and after the baseline and recovery periods using a continuous 0–100 scale (0 = “*not stressed at all*”, 100 = “*very stressed*”). Participants adjusted a slider on a virtual tablet using keyboard keys and verbally confirmed their rating, after which the experimenter manually proceeded to the next phase. Task compliance was assessed by manually recording the duration of the speech during the oral task and noting participant responses during the arithmetic task.

#### Autonomic recordings

During the VR session, we recorded electrocardiogram (ECG), skin conductance (SC), thermal variation, and respiratory rate using a BIOPAC MP35 system linked to a secondary computer running specific software (BIOPAC Student Pro 3.7). The system was configured to sample data at a rate of 500 Hz.

The ECG was recorded using a DI-modified bypass, with Ag/AgCl pre-gelled surface electrodes (BIOPAC EL503, 7% NaCl) placed on the participant’s left and right wrists. The ECG was recorded with a band-pass filter set between 0.5 and 35 Hz.

For SC, we used bipolar Ag/AgCl surface electrodes (BIOPAC EL507) that were pre-gelled with an isotonic electrolyte (0.05 molar NaCl). These electrodes were attached to the palmar side of the middle phalanges of the index and middle fingers of the participant’s non-dominant hand. SC was measured at a gain of 5 µS/V with a 10 Hz low-pass filter.

Temperature variations were recorded using a temperature transducer (BIOPAC SS18LA) affixed to the palmar surface of the ring finger of the participant’s non-dominant hand. These temperature variations were measured at a gain of 2.5°/V with a 0.05 Hz high-pass filter.

Respiration was recorded using a respiratory effort transducer (BIOPAC SS5LB) attached to an adjustable belt on the participants’ thorax. The respiration was recorded with a band-pass filter set between 0.5 and 66.5 Hz.

Skin temperature and respiration were recorded to control for peripheral and respiratory influences on SC and cardiac signals.

### Tasks and procedure

The experiment was conducted between 8:00 a.m. and 12:00 p.m. to minimize circadian influences on autonomic activity and emotional experience^33^. The protocol comprised *three stages* (Fig. 1).

*First*, participants completed the DASS-42 and the LSAS. *Second*, they underwent a stress procedure inspired by the TSST-VR. During this stage, the experimenter remained physically present in the room where the procedure took place to ensure the proper conduct of the session and participant safety, while minimizing any interaction with the participant. This consisted of five consecutive periods of 5 min each: a baseline, two stress-inducing tasks, and their respective recovery periods. The entire procedure took place within the same virtual room. During the baseline and recovery periods, participants were alone in the virtual environment, with no avatars present, and the clock in front of them remained inactive. Instructions specified that participants were allowed to visually explore the environment but were asked to minimize arm movements due to the placement of physiological sensors. The stress periods included an oral presentation task and a mental arithmetic task, delivered in counterbalanced order using block randomization stratified by sex. The task instructions were as follows:

Oral presentation task: “In a moment, you will be faced with a panel of three members from the company STATICA. As part of a job interview for a statistician position, you will be asked to deliver a 5-minute oral presentation. During this time, you should introduce yourself and describe your background, highlighting the qualities that would make you a good candidate for the company. Your performance will be evaluated based on the relevance of your responses and your ability to fully use the allocated time.”

*Mental Arithmetic task*: “In a moment, you will be faced with a panel of three members from the company STATICA. As part of a job interview for a statistician position, you will be asked to perform an arithmetic task for 5 minutes. An auditory signal will indicate the appearance of a three-digit number on the wall clock in front of you. You must then count aloud backwards in steps of seven starting from this number. For example, if a beep indicates the number 423, you should say aloud: 416, 409, 402, 395, and so on. If your responses are too slow, a beep will indicate the presentation of a new starting number, from which you must resume the same task.”

During both tasks, the avatars progressively disengaged from the interaction: the male avatar on the left began checking his phone after 150 s, while the female and second male avatars started conversing with each other after 210 s. During the arithmetic task, new numbers were presented at fixed intervals that progressively decreased from 45 to 15 s. Participants were informed that new numbers would only appear if their responses were too slow, such that the accelerating pace was perceived as negative performance feedback rather than a fixed schedule.

Subjective stress was assessed at the beginning of the baseline and at the end of each period using a slider displayed on the virtual tablet. Participants then received instructions for the next phase and could take as much time as needed to read and understand them before initiating the following period by pressing the space bar on a keyboard.

*Finally*, participants completed the PQ, the CERQ, and the Ricci and Gagnon questionnaires. The entire experiment usually lasted an hour.

### Data analyses

ECG recordings were processed using Kubios HRV Scientific (version 4.1.2.1) for semi-automatic detection of R-waves, followed by manual correction of ectopic beats. A piecewise cubic spline interpolation, set at 4 Hz, was applied to create an equidistantly sampled time series from the non-equidistantly sampled R-R interval data. The R-R intervals were detrended using a smoothness-prior method to remove the slow (< 0.04 Hz) non-stationary trends from the HRV signal. Detected ectopic beats were corrected by replacing the corrupted R-R intervals with interpolated values. Across all participants and recording periods, the mean percentage of R-R intervals replaced by interpolated values following ectopic beat correction was 0.35 ± 0.44% (range: 0.0–3.0%), indicating that the vast majority of the signal reflected true cardiac activity. For the time domain, mean heart rate (HR, in bpm) and RMSSD were computed for each period. For the frequency domain, a power spectrum density analysis was conducted on the R-R interval series using the autoregressive method, from which absolute power (in m.s^2^) of low-frequency variability (LF-HRV, 0.04–0.15 Hz), high-frequency variability (HF-HRV, 0.15–0.4 Hz), and the LF/HF ratio were extracted.

Skin conductance (SC), respiratory rate, and skin temperature were analyzed using AcqKnowledge software version 4.1. For SC, a high-pass filter at 0.05 Hz was applied to isolate the phasic component. Nonspecific skin conductance responses (NS-SCRs) were identified as phasic increases with an amplitude ≥ 0.05 µS^34^, and their frequency (responses per minute, rpm) was computed for each period. All SC signals were visually inspected to identify movement artifacts and non-physiological deflections. Following this systematic inspection, no segments required correction or exclusion. Finally, we calculated the average respiratory rate and skin temperature for each period.

### Statistical analyses

Analyses were conducted in RStudio (v2024.12.1) and figures were created in Python. The significance threshold was set at *p* < .05, with p-values adjusted for multiple comparisons using the Benjamini–Hochberg procedure within each set of related tests (e.g., post hoc comparisons and correlation analyses conducted for each hypothesis).

To test Hypothesis 1, repeated-measures ANOVAs were conducted on subjective stress and autonomic indices, with period (baseline, task 1, recovery 1, task 2, recovery 2) as a within-subject factor. Residual normality was assessed via skewness, kurtosis, and visual inspection; when substantial deviations were observed (e.g., |skewness| or |kurtosis| > 1), Friedman tests were used instead. Sphericity violations were corrected using the Greenhouse–Geisser adjustment (*ε* < 0.75). Significant main effects were followed by planned comparisons between successive periods using paired t-tests or Wilcoxon signed-rank tests, with repeated stress effects assessed by contrasting the reactivity indices between the two tasks. Effect sizes were reported as *ηp²* for ANOVAs, Kendall’s *W* for Friedman tests, Cohen’s *d* for parametric contrasts, and *r* for Wilcoxon tests.

To test Hypothesis 2, we used Pearson correlation coefficients to examine associations between baseline RMSSD and RMSSD and HR levels across periods on the one hand, and their respective reactivity and recovery indices on the other. Reactivity indices were defined as the difference between each task and its preceding period, and recovery indices as the difference between each recovery period and the preceding task; both were averaged across cycles to limit multiple testing. When significant associations were observed, partial correlations controlled for respiratory rate, subjective stress, age, sex, and sense of presence. As a post-hoc complement, Bayesian correlation analyses were conducted to formally quantify evidence for both the null and alternative hypotheses. Bayes factors were computed using the correlationBF function in the BayesFactor package (R) with rscale = 1, a diffuse prior reflecting the absence of strong prior knowledge and the post-hoc, exploratory nature of these analyses. Robustness was verified through sensitivity analyses across a wide range of prior scales (from 0.1 to 2).

To test Hypothesis 3, Pearson correlations were computed to examine associations between baseline RMSSD, subjective stress reactivity, and psychological measures (CERQ, DASS-42, LSAS). Subjective stress reactivity was defined and averaged as described above. Partial correlations controlled for sense of presence when involving subjective stress reactivity, and for respiratory rate and weekly physical activity when involving baseline RMSSD. Post-hoc Bayesian analyses were conducted for associations involving baseline RMSSD, following the same approach described for Hypothesis 2.

As an exploratory analysis, hierarchical multiple regression models were estimated separately for RMSSD reactivity and recovery, entering covariates (respiratory rate, subjective stress, age, sex, sense of presence) in Step 1, psychological distress scores (DASS-42, LSAS) in Step 2, physiological variables (HR changes, baseline RMSSD) in Step 3, and their interaction in Step 4. Regression assumptions were verified and met for all models; diagnostic plots are reported in the Supplementary Materials.

## Results

### Sample characterization

Descriptive statistics for all measures, including means, standard deviations, minima, and maxima for the full sample and by sex, are reported in Supplementary Table S1. Internal consistency was good to excellent for the DASS-42 and LSAS subscales (Cronbach’s α = 0.84–0.93; McDonald’s ω = 0.85–0.93), and good for both adaptive and maladaptive CERQ composite scores (α = 0.80–0.88; ω = 0.80–0.88; see Supplementary Table S2 for detailed indices). At the sample level, DASS-42 scores were within the normal range (depression: M = 6.38, SD = 6.52; anxiety: M = 7.94, SD = 6.66; stress: M = 11.91, SD = 7.21), and the mean LSAS score (M = 42.28, SD = 22.18) was indicative of subclinical social anxiety, confirming that the sample was overall psychologically healthy.

### Paradigm validation: the stress responses to the TSST-VR

**Fig. 2:**
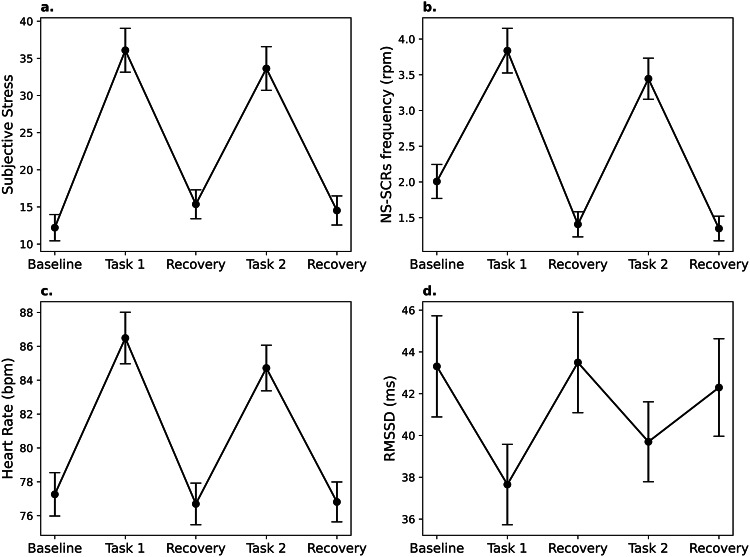
Mean subjective stress level (**a**), non-specific skin conductance responses frequency (NS-SCRs, **b**), heart rate (**c**), and root mean square of the successive differences (RMSSD; **d**) for each period. Error bars represent standard error.

In line with Hypothesis 1, repeated-measures ANOVAs revealed significant period effects on all autonomic and subjective measures, with the exception of HF-HRV (Table 1; Fig. 2). Descriptive statistics for all indices across periods are provided in Supplementary Table S3.

**Table 1:**
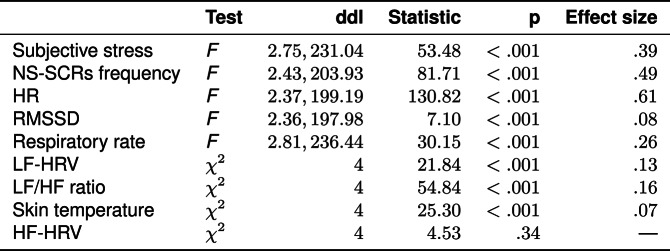
Effect sizes correspond to partial eta squared (*ηp²*) for ANOVAs and Kendall’s *W* for Friedman tests.

Specifically, both stress tasks elicited increases in subjective stress (*ps* < 0.001, *ds* ≥ 0.79), NS-SCRs frequency (*ps* < 0.001, *ds* ≥ 1.00), HR (*ps* < 0.001, *ds* ≥ 1.30), LF-HRV (*ps* ≤ 0.007, *rs* ≥ 0.33), and LF/HF ratio (*ps* < 0.001, *rs* ≥ 0.41), alongside decreases in RMSSD (*ps* ≤ 0.01, *ds* ≤ − 0.28) and respiratory rate (*ps* < 0.001, *ds* ≤ − 0.67). Recovery periods showed the opposite pattern, with significant decreases in subjective stress, NS-SCRs frequency, HR, and LF/HF ratio, and increases in RMSSD and respiratory rate (all *ps* ≤ 0.044). Finally, skin temperature increased during the first task (*p* < .001, *r* = − .57) and the subsequent recovery (*p* = .030, *r* = − .27), then stabilized (*ps* ≥ 0.61, *rs* ≤ 0.14). No differences in reactivity emerged between tasks (*ps* ≥ 0.09).

### The influence of resting HRV on cardiac stress control

**Fig. 3:**
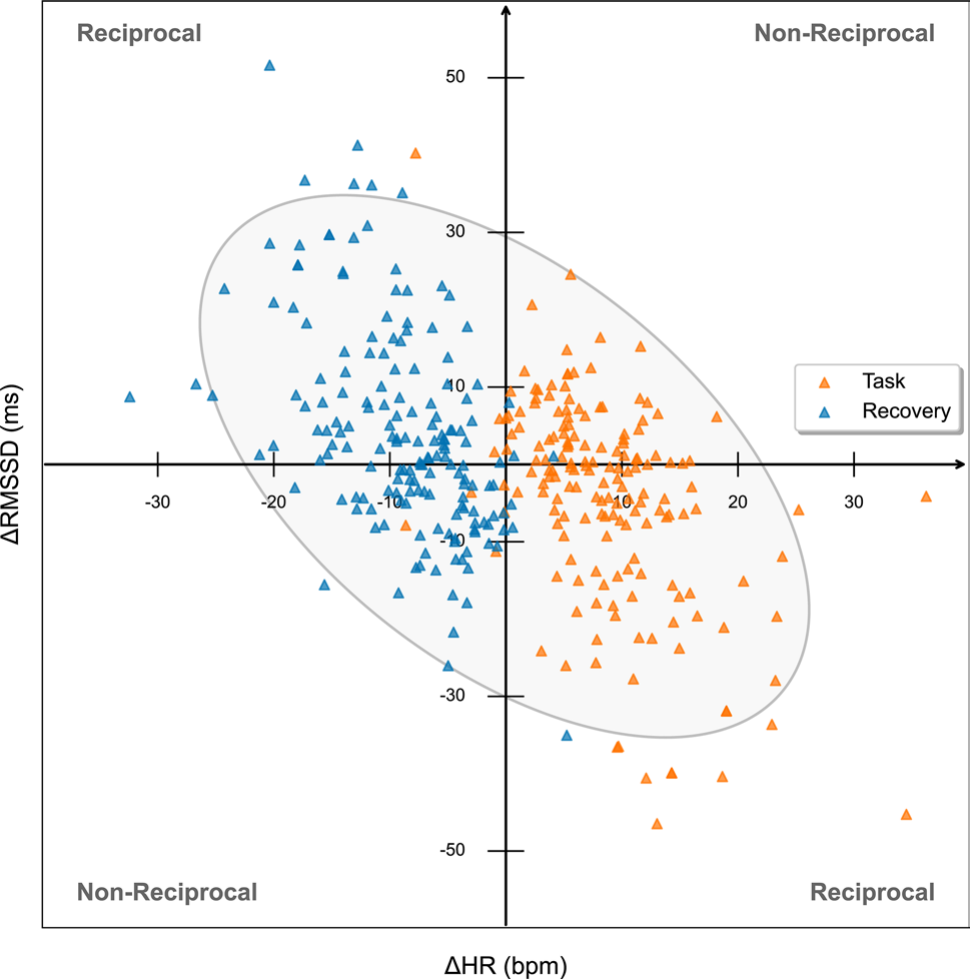
Coordination patterns between HR and RMSSD change across TSST-VR periods. Each point represents the difference between consecutive periods during both tasks (orange) or recovery (blue) periods. The grey ellipse indicates the 95% confidence region of the overall distribution. Data dispersion across the four quadrants reveals diverse autonomic coordination profiles. Reciprocal patterns (upper left and lower right quadrants) account for 58.5% of observations in all periods combined, while non-reciprocal patterns (upper right and lower left) represent 41.5%. This highlights marked interindividual variability in autonomic responses.

To examine Hypothesis 2, we first characterized the variability in the autonomic responses pattern across participants. While HR responses were uniform across participants (96.5% increased during tasks, 97.6% decreased during recovery), RMSSD showed substantial heterogeneity (only 61.9% decreased during tasks, 63.1% increased during recovery). Three coupling patterns emerged between HR and RMSSD changes: reciprocal patterns (opposite-direction changes), uncoupled patterns (HR changes with minimal variation in RMSSD), and non-reciprocal patterns (same-direction changes). Across all periods, reciprocal coordination predominated (58.5%), while alternative patterns (uncoupled and non-reciprocal) collectively accounted for 41.5% of responses (Fig. 3).

We then examined associations between baseline RMSSD and cardiac activity across the protocol. These analyses revealed that the higher the baseline RMSSD, the greater the RMSSD levels (rs ≥ 0.88, ps < 0.001) and the lower the HR levels (rs ≤ − 0.69, ps < 0.001) across all periods. Moreover, baseline RMSSD was associated with greater RMSSD reactivity and recovery (r²s ≥ 0.23, *p* < .001). All these associations remained significant after controlling for respiratory rate, age, sex, and sense of presence (r²s ≥ 0.16, ps < 0.001). In contrast, no significant correlation was found between HR reactivity or recovery and either baseline RMSSD (r²s ≤ -0.00, *p* ≥ .670) or baseline HR (r²s ≤ -0.00, *p* ≥ .670). To further characterize associations between baseline RMSSD and HR changes, we conducted post hoc Bayesian correlation analyses. The results provided moderate evidence in favor of the null hypothesis for both HR reactivity (*r* = .08, *BF₀₁* = 5.76) and HR recovery (*r* = .04, *BF₀₁* = 6.83). Posterior medians were close to zero (HR reactivity: median *r* = .08, 95% CrI [− 0.13, 0.28]; HR recovery: median *r* = .04, 95% CrI [− 0.17, 0.26]), indicating no meaningful association between variables. Sensitivity analyses across a wide range of prior specifications led to qualitatively similar conclusions (HR reactivity: *BF₀₁* range 1.88–8.97; HR recovery: *BF₀₁* range 2.16–10.66).

As an exploratory analysis, hierarchical regression models were used to investigate predictors of RMSSD reactivity and recovery. In both models, each step significantly improved explained variance (Table 2), with physiological variables emerging as the dominant predictors, whereas psychological distress showed negligible contribution.

**Table 2:**
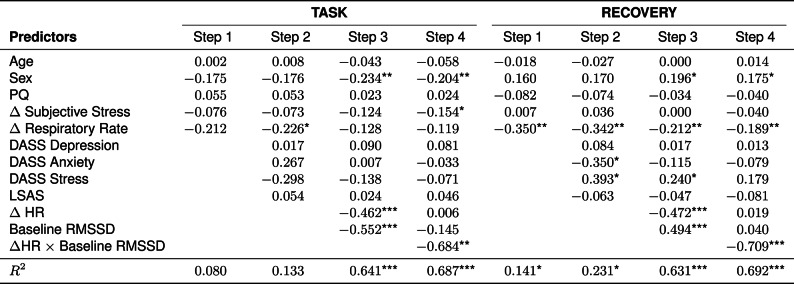
Standardized regression coefficients (*β*) for predictors of RMSSD changes during tasks (relative to preceding periods) and recovery periods (relative to tasks). Models were built in four steps: (1) covariates (respiratory rate, physical activity, age, sex, presence), (2) psychological measures (DASS: Depression, Anxiety, and Stress Scale; LSAS: Liebowitz Social Anxiety Scale), (3) HR change and baseline RMSSD, and (4) interaction term (HR change ×baseline RMSSD). *R²* indicates cumulative variance explained for each model. *p < .05, **p < .01, ***p < .001.

**Fig. 4:**
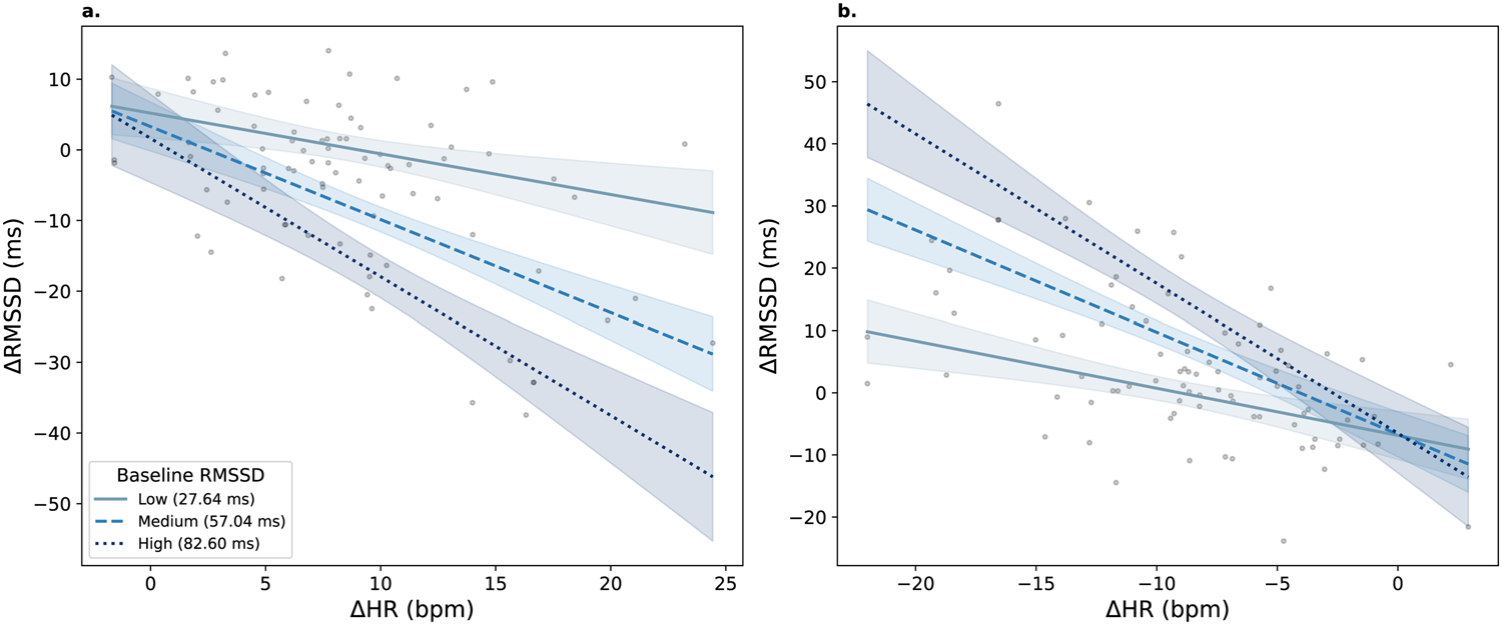
Moderation of the HR–RMSSD reactivity relationship by baseline RMSSD during task (a) and recovery (b) periods. The x- and y-axes show mean changes in HR and RMSSD, respectively (a: tasks minus preceding periods; b: recoveries minus tasks). Individual participants are shown as scatter points, with regression lines indicating the HR–RMSSD relationship for three baseline RMSSD clusters (low: 8.79–40.94 ms, medium: 46.19–66.68 ms, high: 75.06–94.85 ms; see Supplementary Material for clustering procedure). The figure shows that the higher the baseline RMSSD, the more negative the HR–RMSSD relationship becomes during tasks (A; *\:b=-0.024*, *\:p<.001**)*, and recovery (B; *\:b=-0.027*, *\:p<.001**).*

Specifically, Step 1 (covariates) was non-significant for reactivity (*R²* = 0.08, *F(5*,*79)* = 1.37, *p* = .243) but significant for recovery (*R²* = 0.14, *F(5*,*79)* = 2.60, *p* = .031). Compared to Step 1, adding DASS-42 and LSAS scores in Step 2 produced modest but significant increases in variance explained (tasks: Δ*R²* = 0.05, *p* = .026; recovery: Δ*R²* = 0.09, *p* = .001). Including physiological variables in Step 3 (HR changes and baseline RMSSD) yielded substantial improvements (tasks: Δ*R²* = 0.51, *p* < .001; recovery: Δ*R²* = 0.40, *p* < .001). Finally, adding the baseline RMSSD × HR changes interaction in Step 4 further enhanced model fit (tasks: Δ*R²* = 0.05, *p* < .001; recovery: Δ*R²* = 0.06, *p* < .001). The final models both explained 69% of variance (tasks: *R²* = 0.69, *F(12*,*72)* = 13.18, *p* < .001; recovery: *R²* = 0.69, *F(12*,*72)* = 13.49, *p* < .001), with the baseline RMSSD × HR changes interaction emerging as the strongest predictor of RMSSD reactivity and recovery (tasks: *β* = −0.65, *p* = .002, Fig. 4a; recovery: *β* = −0.69, *p* < .001, Fig. 4b).

Finally, to further characterize the baseline RMSSD × HR changes interaction, Johnson–Neyman analyses were conducted. These analyses identified the range of baseline RMSSD values for which the association between HR and RMSSD changes was statistically significant (*p* < .05): above 20.35 ms during tasks and 18.78 ms during recovery, corresponding to 84.7% and 90.6% of participants, respectively. Within these regions, the association between HR and RMSSD changes became increasingly negative as baseline RMSSD increased. Below these thresholds, the association was not statistically significant (tasks: β = −0.44, SE = 0.22, *p* = .05; recovery: β = −0.47, SE = 0.24, *p* = .05), suggesting a weaker and less consistent coupling between HR and RMSSD changes at lower baseline RMSSD levels.

### The influence of resting HRV on stress reactivity and psychological distress

To examine Hypothesis 3, we assessed associations between baseline RMSSD and subjective stress reactivity and psychological variables. No significant association was found between baseline RMSSD and mean subjective stress reactivity (*r* = –.01, *p* = .94). Similarly, baseline RMSSD was not significantly associated with any psychological variable, including adaptive (*r* = − .05, *p* = .705) or maladaptive ER strategies (*r* = − .06, *p* = .659), depression, anxiety, or stress symptoms (*r²s* < 0.01, *ps* ≥ 0.507), or social anxiety (*r* = –.14, *p* = .354). To further characterize these associations, we conducted additional (post-hoc) Bayesian correlation analyses. Results provided moderate evidence in favor of the null hypothesis across all variables, including subjective stress reactivity (*BF₀₁* = 7.33), maladaptive (*BF₀₁* = 6.38) and adaptive ER strategies (*BF₀₁* = 6.65), depressive (*BF₀₁* = 7.34), anxiety (*BF₀₁* = 4.93), and stress symptoms (*BF₀₁* = 7.37), as well as social anxiety (*BF₀₁* = 3.25). Posterior estimates were centered near zero, with median correlations ranging from − 0.14 to 0.01 and 95% credible intervals spanning small negative to small positive values (e.g., − 0.33 to 0.21), indicating no meaningful associations. Sensitivity analyses indicated that these conclusions were robust across a wide range of prior widths (*BF₀₁* range: 1.17–11.51).

We then examined associations between subjective stress reactivity and psychological variables. Subjective stress reactivity was positively associated with symptoms of depression (*r* = .32, *p* = .008), stress (*r* = .29, *p* = .017), and social anxiety (*r* = .37, *p* = .002), all of which survived correction for multiple comparisons. Associations between subjective stress reactivity and anxiety symptoms (*r* = .23, *p* = .076) or maladaptive ER strategies (*r* = .22, *p* = .083) were observed but did not reach significance after correction. All these associations were significant in partial correlations controlling for sense of presence (partial *rs* ranging from 0.22 to 0.37, *ps* ≤ 0.046). No association was observed with adaptive ER strategies (*r* = .08, *p* = .512).

Finally, we examined associations between ER strategies and psychological variables. Maladaptive ER strategies were positively associated with all symptom measures: depression (*r* = .53, *p* < .001), anxiety (*r* = .56, *p* < .001), stress (*r* = .55, *p* < .001), and social anxiety (*r* = .43, *p* < .001), all surviving correction for multiple comparisons. In contrast, adaptive ER strategies showed no significant association with any of these variables (*rs* ranging from − 0.12 to 0.09, *ps* ≥ 0.294). The correlation matrix is available in the supplementary materials (Figure S9).

## Discussion

This study aimed to evaluate the predictive value of HRV as a marker of an individual’s capacity to adaptively respond to stressful situations. Specifically, we examined the association between HRV and cardiac stress responses, and explored whether resting HRV relates to subjective stress reactivity, ER strategies, and psychological distress. To this end, two tasks specifically recognized for generating acute psychosocial stress (TSST) were conducted in a virtual reality (VR) context, in healthy young adults.

Consistent with our hypotheses, the TSST-VR elicited both subjective and autonomic stress responses, supporting the validity of our paradigm. Furthermore, we confirmed that higher baseline RMSSD predicted greater RMSSD reactivity and recovery. As an extension of this finding, exploratory hierarchical regression analyses revealed that the higher the resting RMSSD, the more reciprocal the HR–RMSSD relationship, suggesting that resting HRV predicts individual differences in cardiac vagal control during stress and recovery. However, contrary to our expectations, Bayesian analyses provided evidence in favor of the absence of an association between resting RMSSD and HR reactivity or recovery, subjective stress reactivity, psychological distress, and ER strategies. Instead, frequent use of maladaptive ER strategies was associated with greater psychological distress.

Overall, our findings challenge the view of resting HRV as a marker of adaptive capacity to acute stress or as an index of psychological distress vulnerability. Rather, resting HRV interpretation should be restricted to a predictor of the degree to which the vagal system can modulate cardiac activity, with health relevance likely more tied to long-term cardiovascular vulnerability in the context of repeated or chronic stress.

### Resting HRV predicts cardiac vagal control during stress

In line with previous findings, the TSST-VR successfully elicited subjective and autonomic stress responses across participants^35^, replicating the expected pattern of cardiovascular reactivity at the group level^10^. However, individual trajectories revealed a more complex picture. Since RMSSD reflects parasympathetic inhibitory modulation of cardiac activity^36^, HR and RMSSD would be expected to covary inversely under stress. This reciprocal pattern was indeed predominant, but nearly half of participants displayed alternative coupling patterns. In uncoupled patterns, HR changed while RMSSD remained stable, suggesting that HR variations likely originated from sympathetic influences acting independently from parasympathetic modulation. In non-reciprocal patterns, both metrics changed in the same direction, suggesting that sympathetic and parasympathetic influences acted concurrently rather than in opposition. Although we did not directly measure cardiac sympathetic activity, this interpretation is supported by both empirical findings^5,37^ and the theoretical framework of the autonomic space model^4^. Therefore, in line with this model, our findings challenge the assumption that reciprocal HR–RMSSD coupling represents the typical cardiac response to stress, and instead highlight substantial interindividual heterogeneity in cardiac autonomic control during stress.

In line with our second hypothesis, resting RMSSD predicted greater RMSSD reactivity and recovery. Exploratory analyses extended this finding, showing that resting RMSSD predicted the nature of the HR–RMSSD coupling pattern, with higher resting RMSSD being associated with more reciprocal coordination between these two metrics. Specifically, above a baseline RMSSD threshold of approximately 19–20 ms, higher resting RMSSD was associated with more reciprocal coordination, such that HR changes were accompanied by proportional RMSSD changes. Such a pattern is consistent with a greater capacity of the parasympathetic system to modulate cardiac activity. Below this threshold, the association became non-significant, suggesting that parasympathetic modulation had a less consistent influence on cardiac activity. Such results are consistent with previous findings^37,38^ and support the role of resting HRV as a marker of the parasympathetic system’s capacity to dynamically modulate cardiac activity in response to environmental demands, a capacity that appears to diminish as resting HRV decreases, both during reactivity and recovery. Conversely, the contribution of psychological variables to RMSSD reactivity and recovery was negligible. Although caution is warranted when interpreting this null result, our data does not support the idea that cardiac reactivity under stress can be reliably inferred from an individual’s propensity to experience psychological distress. Importantly, this result contributes to a broader pattern questioning the robustness of the link between HRV and individuals’ capacity to adaptively respond to stressful situations.

### Resting HRV does not predict physiological or psychological adaptive capacity

From a physiological perspective, our analyses provided evidence in favor of the absence of association between resting RMSSD and HR reactivity or recovery. This finding is noteworthy given that stress involves dynamic adjustments of the organism’s state to meet environmental demands, with variations in HR constituting a key component of this process. As such, HR represents a central indicator through which adaptive versus maladaptive autonomic responses to stress would be expected to be expressed. Nonetheless, our findings suggest that HR adjustments were not robustly associated with resting HRV, which challenges the view, commonly put forward in the literature^7,9^, that low HRV is a marker of a reduced capacity for dynamic adaptation.

From a psychological perspective, our analyses similarly provided evidence in favor of the absence of association between resting RMSSD and subjective stress reactivity, ER strategies, and psychological distress. These findings contrast with the widespread view of resting HRV as a robust marker of ER capacity and psychological distress vulnerability^7,39^. This view draws on multiple lines of evidence, notably associations between resting HRV and self-reported ER difficulties^12^, adaptive coping strategies^14,15^, and psychopathology symptoms^39^. However, our findings are not isolated, with other studies similarly failing to observe such correlations^38,40,41^, including meta-analytic work suggesting that these associations are inconsistent and context-dependent^41^. Methodological factors such as reliance on self-report measures and the homogeneous non-clinical nature of our sample may have contributed to this absence of association. Nonetheless, rather than fully accounting for it, such considerations reinforce the view that this relationship is contingent on specific contextual, methodological, and population characteristics.

Taken together, these findings challenge the view of resting HRV as a reliable marker of an individual’s capacity to cope with acute stress or as an index of vulnerability to psychological distress.

### Clinical perspectives

Clinically, although our data do not support using resting HRV to differentiate individuals in their ability to adjust to an acute stressor, they suggest that this marker may help identify individuals at greater cardiovascular risk under repeated or chronic stress. Specifically, resting RMSSD was positively associated with RMSSD levels and negatively associated with HR levels across all periods of the protocol. This indicates that individuals with lower resting HRV exhibit consistently elevated HR throughout stress and recovery. Importantly, elevated HR and reduced HRV are established risk factors for all-cause mortality^42,43^. Hence, low resting HRV reflect an unfavorable physiological profile that may contributes to sustained cardiovascular strain under repeated or chronic stress.

Alongside these autonomic findings, our results indicate that frequent reliance on maladaptive ER strategies was associated with greater overall psychological distress and heightened subjective stress reactivity, although this latter association did not survive correction for multiple comparisons. This pattern suggests that ER strategies may play a role in shaping individuals’ emotional experiences, with greater use of maladaptive strategies potentially corresponding to more frequent and intense negative affect. This interpretation is consistent with prior evidence that psychological vulnerability stems more from the excessive use of maladaptive ER strategies than from a lack of adaptive ones^17^. Given that subjective stress is typically accompanied by cardiovascular activation, frequent use of maladaptive ER strategies would likely exert greater strain on individuals with low resting HRV compared to those with higher HRV. This hypothesis is consistent with recent evidence showing that lower resting HRV strengthens the association between expressive suppression, a specific form of maladaptive ER strategy, and depressive symptoms^44^. Consequently, the combination of low resting HRV and frequent maladaptive ER strategies likely increase the vulnerability to stress-related disorders.

### Methodological implications

Methodologically, our findings lend strong support to greater integration of the autonomic space model into stress and HRV research. The reciprocity doctrine, which assumes that increases in sympathetic activity are necessarily accompanied by decreases in parasympathetic activity and vice versa, remains pervasive in the field. This persistence both undermines the validity of current findings and perpetuates misconceptions that shape experimental design and data interpretation.

Firstly, HR and HRV variations during stress are often expected to follow a stereotyped and inversely coupled pattern, making them interchangeable markers of stress reactivity. Our findings, along with others, challenge this assumption^5,37^. Indeed, as shown in our study, HRV reactivity may increase, decrease, or remain stable during stress even in healthy populations. Moreover, unlike HR, RMSSD does not directly influence cardiac output adjustments to meet environmental demands. Consequently, HRV reactivity should not be considered a reliable marker of stress, whereas HR alone provides an adequate metric for group-level stress assessment.

*Secondly*, the most serious pitfall of the reciprocal model is that it acknowledges only one normative pattern of HR and HRV reactivity to stress and recovery, implicitly defining any deviation as abnormal. Yet, as our findings demonstrate, variability in HRV reactivity is common even among healthy individuals because it is largely determined by resting HRV, which itself shows substantial inter-individual variability. This variability poses a major methodological challenge for clinical research, where group comparisons are commonly used to identify markers of pathology. Given that numerous studies have reported lower resting HRV across various pathological conditions^13^, reduced RMSSD reactivity in patient groups should not be interpreted as evidence of autonomic dysfunction but rather as a predictable consequence of lower resting values. Notably, these lower resting values may reflect genuine pathophysiological alterations but can also stem from confounding factors such as random sampling variation, lifestyle differences^29^, medication use^45^, or cumulative effects of chronic stress exposure^46^. Furthermore, relying on conventional statistical tests of baseline differences to justify subsequent reactivity comparisons is insufficiently robust and potentially misleading. Non-significant results do not constitute evidence of equivalence but merely indicate insufficient evidence for a difference. Indeed, non-rejection of the null hypothesis is particularly likely under two common conditions: small sample sizes, typical of psychophysiological research^47^, and high within-group variability, which is inherent to resting HRV. Consequently, future research comparing clinical groups should either limit interpretations to resting levels or implement more robust methods to control for baseline differences, such as applying inclusion or stratification criteria based on resting RMSSD, or modeling baseline values as covariates or random effects in mixed-model analyses. Alternatively, RMSSD is better suited for research designs that capitalize on individual variability rather than attenuate it, such as correlational analyses, person-centered approaches, or latent profile analyses examining distinct autonomic response patterns.

### Strengths and limitations

This study presents several methodological strengths. First, the use of virtual reality enabled highly standardized stress induction while maintaining ecological validity and reducing administration costs. The TSST-VR has been shown to reliably elicit subjective, autonomic and hormonal stress responses comparable to traditional in-vivo protocols^20,35^, validating VR as a versatile tool for stress research. This approach opens promising avenues for future investigations, as VR environments can be easily adapted to expose participants to diverse, ecologically relevant stressors while preserving experimental control. Second, we controlled for numerous potential confounds, including respiratory rate, sex, and sense of presence. Importantly, our sample’s homogeneity, young, healthy, and physically active adults, provided additional control over age and lifestyle factors that substantially influence resting HRV, allowing clearer isolation of resting HRV’s predictive value for stress regulation and psychological distress.

In terms of limitations, the previous methodological advantage in isolating baseline HRV effects comes at the cost of limited generalizability to broader populations, particularly clinical groups and older adults. In addition, the cross-sectional design further precludes inferences about long-term health trajectories. Longitudinal research is therefore needed to determine whether low resting HRV prospectively predicts the onset of stress-related cardiovascular and psychological disorders. Finally, the absence of direct cardiac sympathetic measures limits our ability to fully characterize autonomic coordination patterns. Although we recorded skin conductance, the autonomic nervous system is not unitary^48^, hence electrodermal activity cannot substitute for cardiac-specific indices^49^. Future research should incorporate measures such as the pre-ejection period to provide more conclusive evidence for the autonomic space model.

## Conclusion

This study examined the predictive value of resting HRV as a marker of an individual’s capacity to respond adaptively to stressful situations, both physiologically and psychologically. Three key findings emerged: (1) higher resting HRV predicted greater parasympathetic modulation of cardiac activity during stress and recovery; (2) Bayesian analyses provided evidence in favor of the absence of an association between resting RMSSD and HR reactivity or recovery, subjective stress reactivity, psychological distress, and ER strategies; (3) frequent use of maladaptive ER strategies was associated with greater psychological distress.

These findings carry theoretical, methodological, and clinical implications. Theoretically, they challenge the view that resting HRV indexes individual differences in adaptive capacity. This apparent association in previous literature may reflect uncontrolled lifestyle confounds and an overreliance on the reciprocity model, which conflates normal inter-individual variability in autonomic patterns with dysregulation. Methodologically, HRV reactivity may increase, decrease, or remain stable during stress even in healthy populations. Unlike HR, it should therefore not be treated as a reliable stress marker. Clinically, low resting HRV may reflect an unfavorable autonomic profile that amplifies cardiovascular strain under chronic stress. Individuals who frequently rely on maladaptive ER strategies are more likely to experience recurrent negative affect, thereby increasing their exposure to chronic stress. The co-occurrence of both risk factors may therefore delineate a particularly vulnerable profile for stress-related disorders. Given the apparent independence between these two dimensions, their joint systematic assessment represents a promising avenue for prevention and intervention in physical and mental health.

## Electronic Supplementary Material

Below is the link to the electronic supplementary material.

Supplementary Material 1

## Data Availability

The datasets analyzed during the current study are available from the corresponding author on request.
