# Supplementary material for: Resting HRV predicts cardiac vagal control during stress, not psychological distress
Source: Sci Rep. 2026 May 23;16:23670. doi: 10.1038/s41598-026-52956-z (PMC13424317; doi:10.1038/s41598-026-52956-z)
Supplement: Supplementary file 1 — Supplementary Material 1 [file 41598_2026_52956_MOESM1_ESM.pdf]

Supplementary Data

1. Sample characteristics

	**Women**	**Men**	**Total**	*Min–Max*
Age	21.72 (2.78)	22.14 (3.27)	21.92 (3.03)	18–34
Education level (ISCED)	5.29 (1.79)	5.57 (1.38)	5.68 (1.28)	3–8
DASS depression	5.56 (6.65)	7.21 (6.35)	6.38 (6.52)	0–30
DASS anxiety	8.00 (7.05)	7.88 (6.31)	7.94 (6.66)	0–30
DASS stress	12.16 (8.18)	11.64 (6.16)	11.91 (7.21)	0–36
LSAS	46.49 (22.04)	37.98 (21.75)	42.28 (22.18)	0–103
CERQ adaptative	67.42 (12.70)	68.04 (11.53)	67.73 (12.07)	39–100
CERQ maladaptative	38.58 (7.58)	36.80 (8.47)	37.31 (8.09)	16–57
Ricci & Gagnon	22.93 (5.12)	24.19 (5.67)	23.55 (5.41)	11–35
Sense of presence	86.60 (15.45)	86.62 (16.98)	87.60 (16.16)	42–134

Table S1. Descriptive characteristics of the sample. Mean (Standard Deviation).

2. Internal consistency of psychometric measures

**Scale**	**Items**	**Cronbach's α**	**McDonald's ω**
DASS Depression	14	.898	.908
DASS Anxiety	14	.838	.849
DASS Stress	14	.852	.861
LSAS Anxiety	24	.927	.928
LSAS Avoidance	24	.863	.862
CERQ Adaptive	20	.875	.878
CERQ Maladaptive	16	.798	.803
Presence – Realism	7	.827	.830
Presence – Action	4	.580	.598
Presence – Interface	3	.495	.509
Presence – Examine	3	.394	.553
Presence – Performance	2	.649	.676

Table S2. Internal consistency of the main psychometric measures assessed in the present sample.

3. Stress response to the TSST-VR

	**Baseline**	**Task1**	**Recovery1**	**Task2**	**Recovery2**
Subjective stress	12.21 (16.30)	36.09 (27.18)	15.36 (17.94)	33.64 (27.13)	14.53 (18.08)
NS-SCRs frequency	2.01 (2.20)	3.84 (2.88)	1.41 (1.62)	3.44 (2.65)	1.35 (1.58)
HR (bpm)	77.26 (11.81)	86.49 (14.01)	76.70 (11.32)	84.72 (12.40)	76.81 (10.86)
RMSSD (ms)	43.31 (22.31)	37.65 (17.73)	43.50 (22.15)	39.70 (17.64)	42.30 (21.52)
LF (ms^2)	1371.68 (1191.72)	1810.87 (1247.86)	1682.87 (1391.11)	1976.69 (1138.52)	1837.36 (1467.53)
HF (ms^2)	1010.62 (1055.56)	778.60 (665.88)	981.10 (925.30)	851.80 (755.92)	928.77 (917.83)
LF/HF ratio	2.44 (2.74)	3.10 (1.83)	2.64 (2.39)	3.20 (1.83)	3.13 (3.31)
SDNN (ms)	48.45 (18.60)	50.58 (17.95)	51.28 (19.69)	53.49 (16.91)	52.28 (20.16)
Skin temperature (℃)	29.47 (4.09)	30.18 (4.17)	30.40 (4.23)	30.41 (4.16)	30.40 (4.19)
Respiration rate (rpm)	14.06 (2.41)	11.98 (1.86)	13.41 (2.36)	11.74 (2.18)	12.96 (2.50)

Table S3. Physiological and Subjective Measures Across Experimental Periods. Mean (Standard Deviation).

4. Variability in HRV reactivity

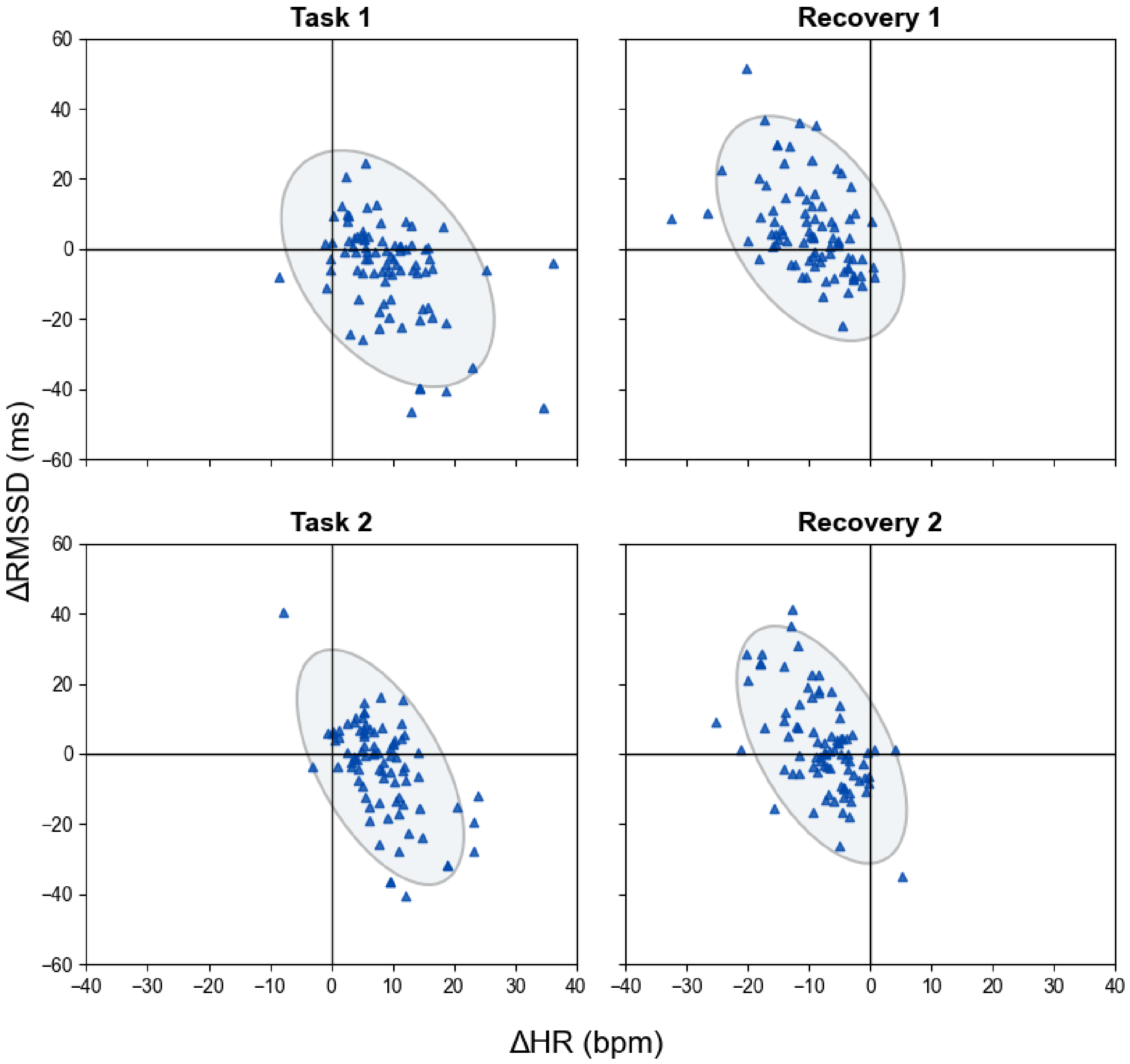

Figure S1. Period-to-period changes in cardiac autonomic responses across TSST-VR phases. Each panel represents a distinct experimental period, with individual data points indicating changes between consecutive phases. Grey ellipses denote the 95% confidence region of the overall distribution. Within each panel, the spread of data across the four quadrants reflects diverse autonomic coordination modes — from reciprocal regulation (upper left and lower right quadrants) to non-reciprocal patterns (upper right and lower left quadrants) — underscoring substantial inter-individual variability in stress response dynamics.

5. Hierarchical Multiple Regression Models

5.1. Task Periods

5.1.1. Outlier Detection (Grubbs' Test)

G = 3.05, U = 0.89, p = .075
No significant outlier was detected (α = .05).

5.1.2. Homoscedasticity (Breusch–Pagan Test)

BP = 20.234, df = 12, p = .063
No significant heteroscedasticity detected (α = .05).

5.1.3. Independence of Residuals (Durbin–Watson Test)

DW = 1.96, p = .898
No significant autocorrelation of residuals detected (α = .05).

5.1.4. Linearity (Residuals vs Fitted Plot)

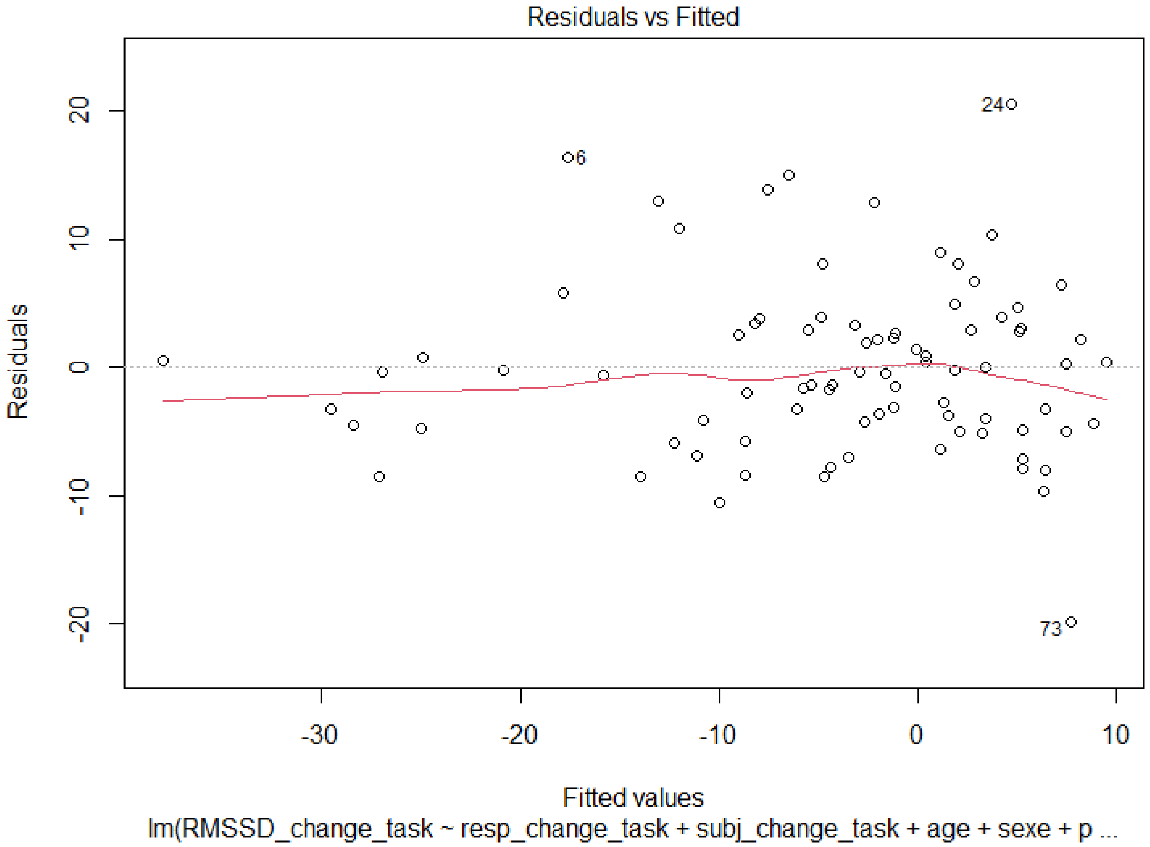

Figure S2. Residuals vs Fitted plot for Model 4 (task phase). Residuals are randomly scattered around zero, indicating no major violation of the linearity assumption.

5.1.5. Normality of Residuals (Q–Q Plot)

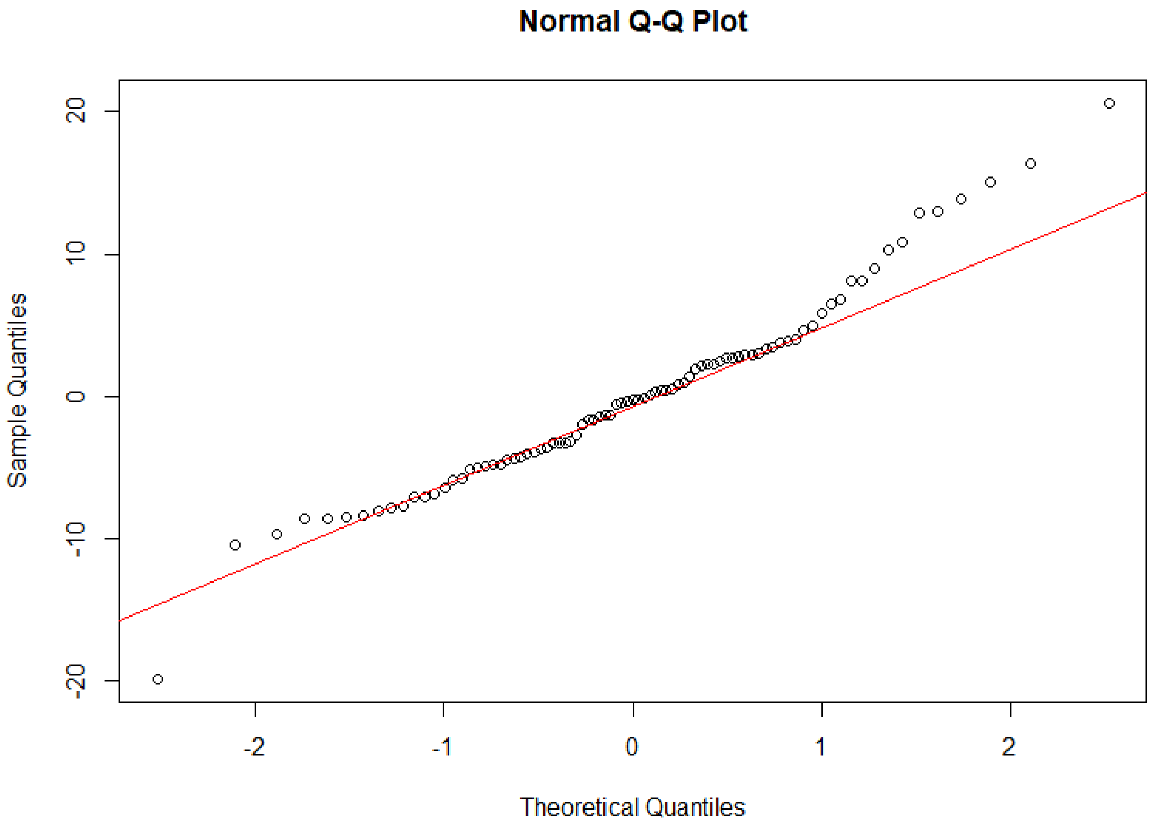

Figure S3. Q–Q plot of residuals for Model 4 (task phase). Residuals closely follow the theoretical quantile line, indicating no major deviation from normality.

5.1.6. Hierarchical multiple regression

Model	Res.Df	RSS	ΔDf	Sum of Sq	F	p
1	79	11268.3	–	–	–	–
2	75	10618.6	4	649.6	3.05	.022 *
3	73	4395.1	2	6223.5	58.46	<.001 ***
4	72	3832.3	1	562.8	10.57	.002 **

Note. . $p < .10$, * $p < .05$, ** $p < .01$, *** $p < .001$.

Table S5. Hierarchical regression model comparison (task phase)

5.2. Recovery Periods

5.2.1. Outlier Detection (Grubbs' Test)

5.2.2. Outlier Detection (Grubbs' Test)

G = 2.81, U = 0.90, p = .173
No significant outlier was detected (α = .05).

5.2.3. Homoscedasticity (Breusch–Pagan Test)

BP = 14.52, df = 12, p = .269
No significant heteroscedasticity detected (α = .05).

Predictor	Slope (B)	β	t	p	VIF
Intercept	15.00	—	1.81	.074	—
Age	-0.23	-0.06	-0.86	.395	1.08
Sex	-4.90	-0.20	-2.88	.005**	1.16
PQ	0.02	0.02	0.33	.741	1.24
Δ Subjective Stress	-0.10	-0.15	-2.01	.048*	1.35
Δ Respiratory Rate	-0.68	-0.12	-1.68	.097	1.15
DASS Anxiety	-0.06	-0.03	-0.28	.782	3.19
DASS Depression	0.15	0.08	0.83	.412	2.23
DASS Stress	-0.12	-0.07	-0.68	.502	2.53
LSAS	0.02	0.05	0.54	.590	1.63
Δ HR	0.01	0.01	0.04	.969	5.88
Baseline RMSSD	-0.08	-0.15	-1.02	.311	4.65
ΔHR $\times$ Baseline RMSSD	-0.02	-0.68	-3.25	.002**	10.18
Residual SE = 7.30 $R^2 = .64$ $F(12,72) = 13.18$, $p < .001$					

Note. † $p < .10$, * $p < .05$, ** $p < .01$, *** $p < .001$.

Table S4. Hierarchical multiple regression predicting RMSSD change during task (Model 4). Abbreviations: PQ = Presence Questionnaire; DASS = Depression Anxiety and Stress Scale; LSAS = Liebowitz Social Anxiety Scale; HR = heart rate; RMSSD = root mean square of successive differences. Δ indicates change scores calculated as task minus prior period.

5.2.4. Independence of Residuals (Durbin–Watson Test)

DW = 2.27, *p* = .198
No significant autocorrelation of residuals detected (α = .05).

5.2.5. Linearity (Residuals vs Fitted Plot)

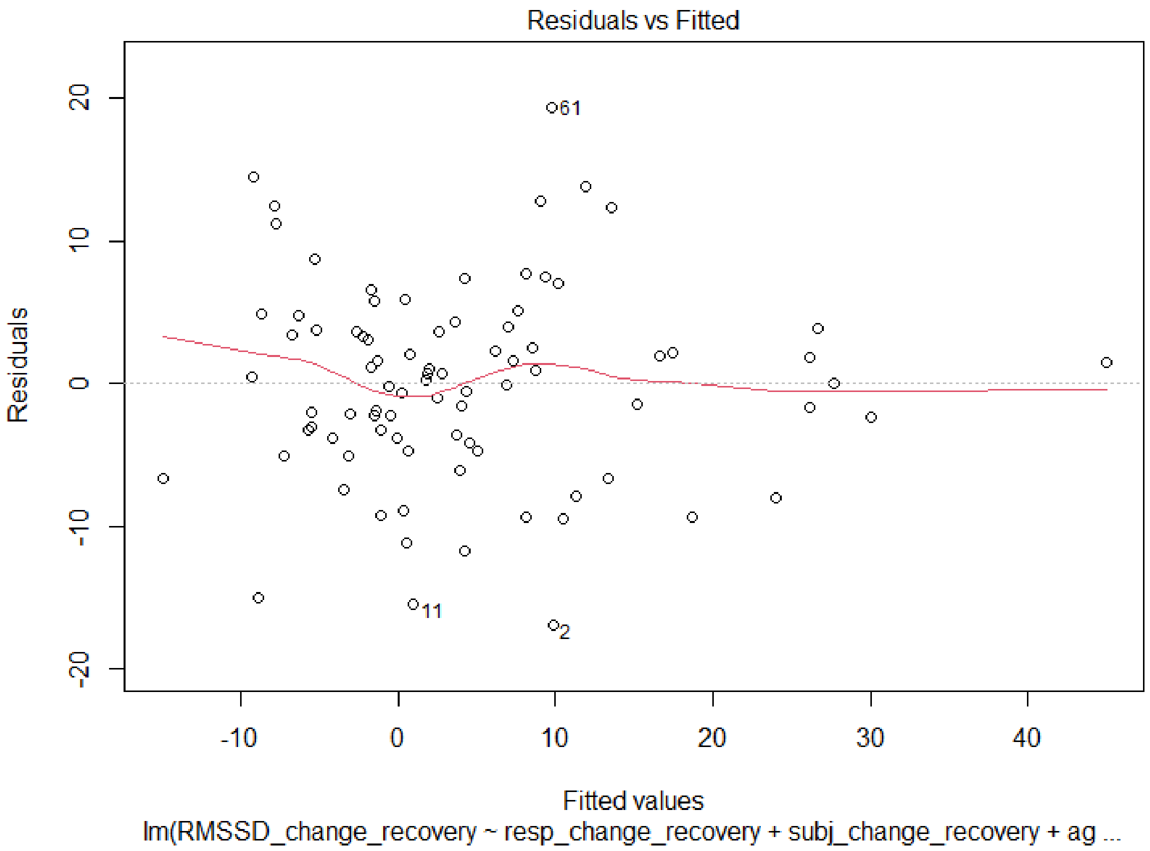

Figure S4. Residuals vs Fitted plot for Model 4 (recovery phase). Residuals are randomly scattered around zero, indicating no major violation of the linearity assumption.

5.2.6. Normality of Residuals (Q–Q Plot)

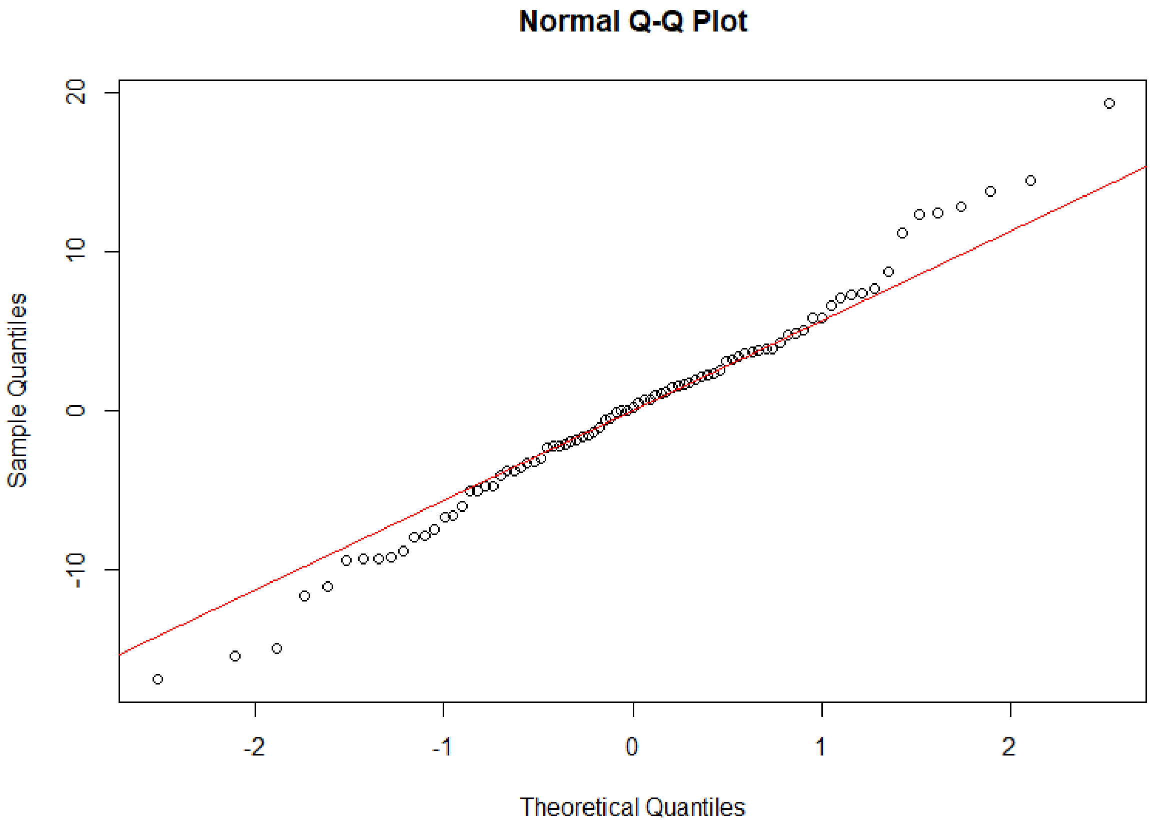

Figure S5. Q–Q plot of residuals for Model 4 (recovery phase). Residuals closely follow the theoretical quantile line, indicating no major deviation from normality.

5.2.7. Hierarchical multiple regression

Predictor	Slope (B)	β	t	p	VIF
Intercept	-7.35	—	-0.87	.389	—
Age	0.06	0.01	0.21	.836	1.07
Sex	4.32	0.18	2.51	.014*	1.14
PQ	-0.03	-0.04	-0.55	.587	1.26
Δ Subjective Stress	-0.03	-0.04	-0.55	.587	1.28
Δ Respiratory Rate	-1.05	-0.19	-2.70	.009**	1.15
DASS Depression	0.02	0.01	0.13	.899	2.26
DASS Anxiety	-0.15	-0.08	-0.69	.496	3.10
DASS Stress	0.31	0.18	1.76	.082†	2.42
LSAS	-0.05	-0.08	-1.01	.318	1.53
Δ HR	0.04	0.02	0.13	.900	5.05
Baseline RMSSD	0.02	0.04	0.29	.773	4.42
ΔHR $\times$ Baseline RMSSD	-0.03	-0.71	-3.79	.0003***	8.18
Residual SE = 7.43	$R^2 = .69$	Adj. $R^2 = .64$	$F(12, 72) = 13.49, p < .001$		

Note. † $p < .10$, * $p < .05$, ** $p < .01$, *** $p < .001$.

Table S6. Hierarchical multiple regression predicting RMSSD change during recovery (Model 4). Abbreviations: PQ = Presence Questionnaire; DASS = Depression Anxiety and Stress Scale; LSAS = Liebowitz Social Anxiety Scale; HR = heart rate; RMSSD = root mean square of successive differences. Δ indicates change scores calculated as recovery minus task.

Model	Res.Df	RSS	ΔDf	Sum of Sq	F	p
1	79	11100.0	–	–	–	–
2	75	9945.5	4	1154.5	5.22	$<$.001 ***
3	73	4773.8	2	5171.7	46.79	$<$.001 ***
4	72	3979.4	1	794.3	14.37	$<$.001 ***

Note. . $p < .10$, * $p < .05$, ** $p < .01$, *** $p < .001$.

Table S7. Hierarchical regression model comparison (recovery phase)

6. Clustering Methodology for Baseline RMSSD Visualization

To facilitate the visualization of how baseline cardiac autonomic activity modulates the HR - RMSSD reactivity coupling presented in Figure 4 of the main manuscript, participants were grouped into three illustrative categories based on their resting RMSSD values. This clustering served purely descriptive purposes to enhance the interpretability of the moderation effect and was not used for statistical inference.

Grouping was performed using k-means clustering on baseline RMSSD values, with the optimal number of clusters (k = 3) determined using the elbow method (Figure S6). This approach yielded three distinct groups with minimal overlap: Cluster 1 (low baseline RMSSD) comprised 52 participants with resting RMSSD values ranging from 8.79 to 40.94 ms; Cluster 2 (medium baseline RMSSD) included 20 participants with values between 46.19 and 66.68 ms; and Cluster 3 (high baseline RMSSD) consisted of 13 participants with values spanning 75.06 to 94.85 ms (Figure S7).

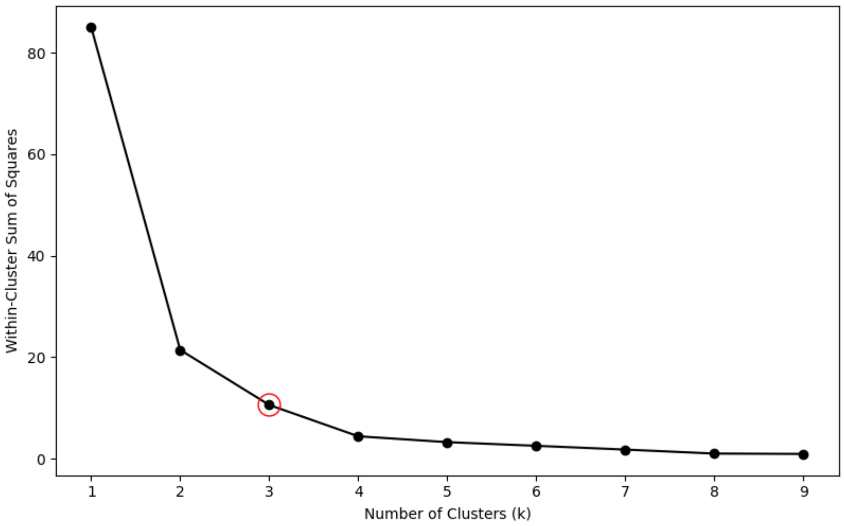

Figure S6. Elbow method for determining the optimal number of clusters. The within-cluster sum of squares is plotted as a function of the number of clusters (k). The elbow point at k = 3 (indicated by the red circle) represents the optimal balance between model parsimony and within-cluster homogeneity, showing diminishing returns in variance reduction beyond three clusters.

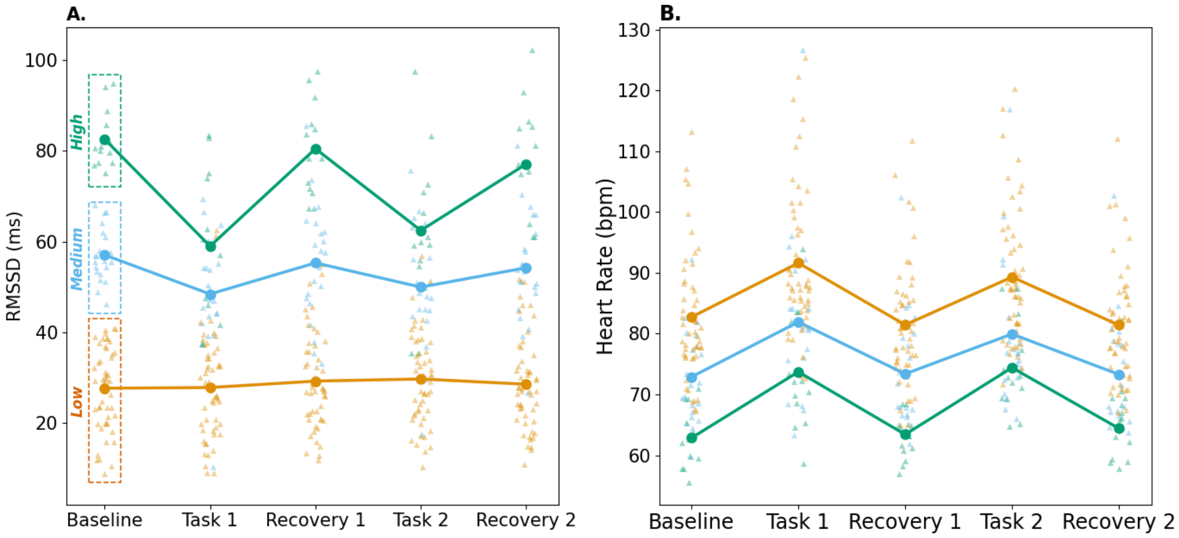

Figure S7. RMSSD (A) and heart rate (B) dynamics across experimental periods, segmented by resting RMSSD-based cluster groups. Clusters were defined using k-means analysis on baseline RMSSD values: low (orange, $<$ 41 ms), medium (blue, 46.19–66.68 ms), and high (green, $>$ 75 ms). Colored rectangles indicate each participant's baseline classification; triangles represent individual trajectories. Lines show average RMSSD and HR trends per group. The figure illustrates how baseline HRV shapes both absolute levels and temporal changes in autonomic cardiac activity.

7. Johnson-Neyman analyses

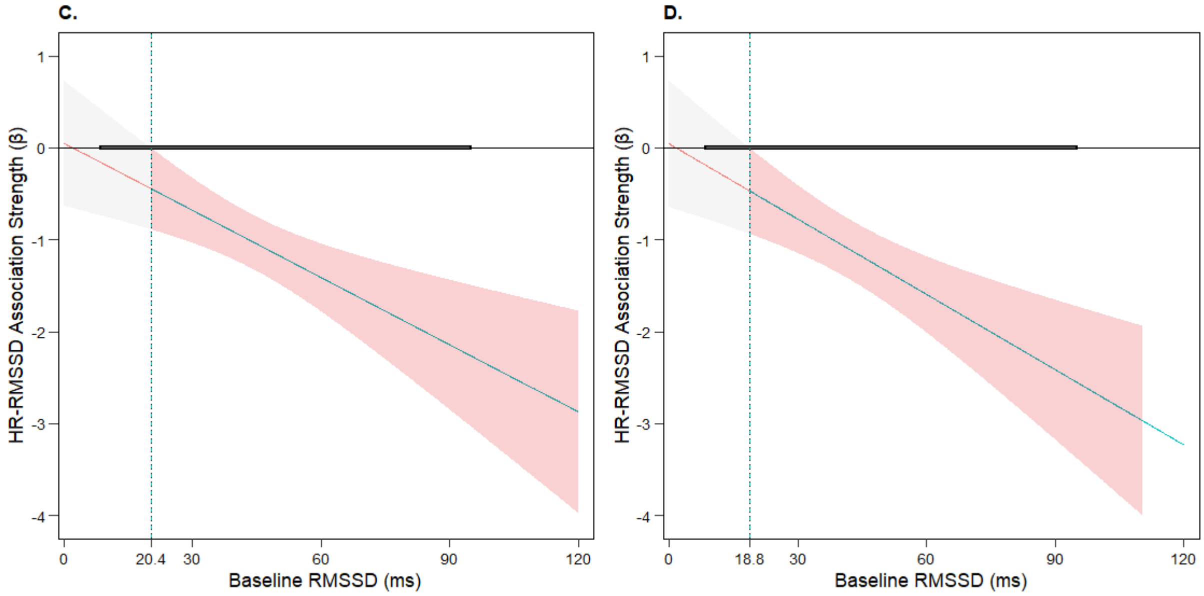

Figure S8. Johnson-Neyman intervals for the moderation of the HR–RMSSD reactivity relationship by baseline RMSSD during task (C) and recovery (D) periods. The y-axis shows the conditional slope β of HR change predicting RMSSD change at each level of baseline RMSSD (x-axis). Shaded bands represent 95% confidence intervals. Vertical dashed lines mark the Johnson-Neyman significance thresholds (task, C: 20.35 ms; recovery, D: 18.78 ms), below which the HR–RMSSD relationship becomes non-significant ($p > .05$).

8. Correlation Matrix of Psychological Variables

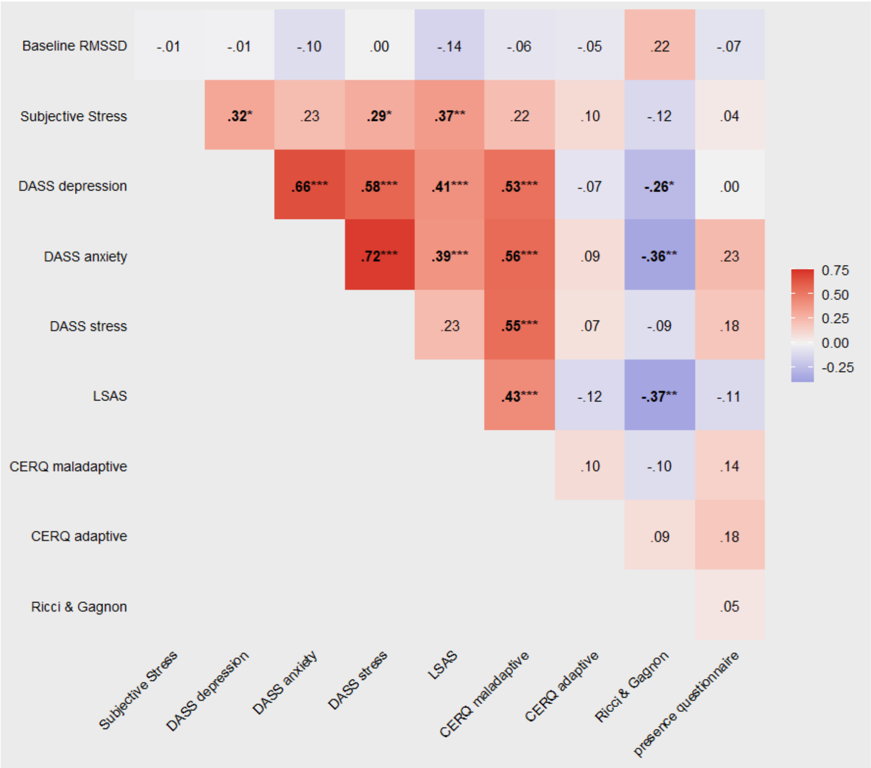

Figure S9. Correlation matrix including baseline RMSSD, subjective stress reactivity, and psychological variables (DASS depression, anxiety, stress, LSAS, CERQ subscales, Ricci & Gagnon, and Presence Questionnaire). Pearson correlation coefficients are displayed, with color intensity reflecting the strength and direction of associations. Asterisks indicate significance levels (* $p < .05$, ** $p < .01$, *** $p < .001$).
